# The association between dietary amino acids and the risk of nonalcoholic fatty liver disease among Tehranian adults: a case-control study

**DOI:** 10.1186/s40795-022-00656-y

**Published:** 2022-12-27

**Authors:** Ebrahim Mokhtari, Hamid Ahmadirad, Farshad Teymoori, Azadeh Mohammadebrahim, Samaneh Sadat Bahrololomi, Parvin Mirmiran

**Affiliations:** 1 Student Research Committee, Nutrition and Endocrine Research Center, Research Institute for Endocrine Sciences, Shahid Beheshti University of Medical Sciences, Tehran, Iran; 2 Nutrition and Endocrine Research Center, Research Institute for Endocrine Sciences, Shahid Beheshti University of Medical Sciences, P.O. Box: 1985717413, Tehran, Iran; 3 Department of Nutrition, School of Public Health, Iran University of Medical Sciences, Tehran, Iran; 4 Department of Nutrition, health and treatment center of shahriyar, Iran University of Medical Sciences, Tehran, Iran

**Keywords:** Amino acids, NAFLD, BCAA, Diet, Iran

## Abstract

**Background:**

Amino acids (AAs) are important bioactive components in the diet that can be involved in various underlying biological processes that contribute to the development of nonalcoholic fatty liver disease (NAFLD). The present study investigates the association between dietary intake of amino acids and NAFLD in Iranian adults.

**Methods:**

This study was conducted among 225 newly diagnosed cases of NAFLD and 450 controls. A valid and reliable 168-item semiquantitative food frequency questionnaire (FFQ) was used to collect participants’ dietary intakes. Multivariable logistic regression models were used to assess the association between tertiles of branched-chain amino acids (BCAAs), aromatic amino acids (AAAs), and sulfuric amino acids (SAAs) intake with the odds of NAFLD among the study participants.

**Results:**

The mean ± standard deviation of age and BMI of participants (53% male) were 38.1 ± 8.8 years and 26.8 ± 4.3 kg/m2, respectively. In the final models, the OR and 95% CI of NAFLD among participants in the highest tertiles of BCAAs, AAAs, and SAAs intake compared with those in the lowest tertiles were (OR = 2.82; 95% CI: 1.50–5.30), (OR = 2.82; 95% CI: 1.50–5.30), (OR = 2.86; 95% CI: 1.49–5.48), respectively.

**Conclusion:**

Our study indicated a direct association between the intake of AAs groups, including BCAAs, AAAs, SAAs, and the odds of NAFLD. We suggest that other researchers examine the association between AAs groups and NAFLD in large cohort studies.

## Background

Nonalcoholic fatty liver disease is one of the most common liver-related disorders, affecting about 25% of the global population [1]. The disease is defined as an unusual accumulation of fat in liver cells (more than 5% of cell weight) in a person who does not consume alcohol or consumes less than 20 g per day and has no history of viral hepatitis [2, 3]. NAFLD has multifactorial pathogenesis and is closely linked with other metabolic disorders. So, recently, Buzzetti E et al. suggested a hypothesis called the “multiple-hit” theory to explain complex contractions between several factors, finally leading to NAFLD development, including diet, insulin resistance (IR), adipokines, the intestinal microbiome, genetic and epigenetic factors [4].

According to the theory mentioned above, diet is considered the most important modifiable factor that can either initiate and worsen the NAFLD development process or prevent it and control its complications. Insulin resistance, inflammation, and oxidative stress are key mediating factors in the metabolic dysfunction underlying NAFLD pathogenesis, directly affected by dietary habits. Amino acids are important bioactive components in the diet that can be involved in various underlying biological processes that contribute to the development of NAFLD. However, human studies in this context are limited, and most of them have been conducted on rodent models or in-vitro [5]. Specific dietary amino acids have been shown to affect NAFLD pathogenesis, including glucose metabolism and homeostasis, inflammatory processes, and integrity of the intestinal epithelial barrier [5]. Branched-chain amino acids (BCAAs) (including leucine, isoleucine, and valine) may be the best-known amino acids, contracting with liver metabolic processes. Previous studies demonstrated that a damaged upregulation of the tricarboxylic acid (TCA) cycle, mediated by BCAAs, can lead to mitochondrial dysfunction in NAFLD [6]. Studies conducted in animals and humans showed that supplementation of BCAAs may ameliorate liver steatosis in individuals with nonalcoholic steatohepatitis (NASH)-related liver cirrhosis [7–9].

Since NAFLD is known as a hepatic manifestation of metabolic syndrome, the impact of amino acids on this disease and its component may affect NAFLD development. In this regard, Asghari et al., in a prospective cohort, demonstrated that a higher intake of BCAAs, especially leucine and valine, increases the risk of IR in adults. This study has not found any significant relationship between BCAAs intake and hyperinsulinemia, β-cell dysfunction, or insulin insensitivity [10]. Also, a review study claimed that strong evidence indicates that chronic increases in serum levels of BCAAs causally lead to insulin resistance development [11]. In contrast, a cross-sectional study among Brazilian adults found an inverse association between dietary intakes of BCAAs, particularly leucine, and total cholesterol (TC)/high-density lipoprotein cholesterol (HDL-C) and triglyceride (TG)/HDL-C ratios [12]. Teymoori et al. revealed that some dietary amino acids could potentially increase or decrease serum lipid profile [13]. Also, another study showed that some dietary amino acids related to serum TG, HDL-C, and TC [14]. A previous study showed a direct relationship between dietary sulfuric amino acids (SAAs) with a higher prevalence of Overweight/Obesity, BMI and waist circumference, unfavorable lipid profiles, and IR in Northern Chinese adults [15]. Results about aromatic amino acids (AAAs) are inconclusive. A recent animal study suggested that AAAs have the potential to ameliorate steatosis [16]. However, some investigations have found increased AAAs in liver disease [17].

Therefore, as can be deduced from the literature mentioned above, human studies on the relationship between dietary amino acid intake and the risk of NAFLD are limited. However, animal and laboratory studies have examined this relationship. There is also considerable evidence for a link between dietary amino acids and fatty liver risk factors such as unfavorable lipid profile and insulin resistance. So, the present study aims to investigate the association between dietary intake of amino acids and NAFLD in adults in a case-control design.

## Materials and methods

### Study population

This study was conducted in the metabolic liver disease research center with a case-control design, and we selected patients with convenience sampling. 225 newly diagnosed cases of NAFLD and 450 controls, aged 20–60 years, were enrolled in this study. NAFLD was identified by the non-consumption of alcohol and other causes of liver disease, and an ultrasonography scan of the liver was consistent with NAFLD. An experienced physician did ultrasonography implementation and analysis. The control group was selected based on liver ultrasonography from healthy individuals.

The inclusion criteria for this study were lack of specific diet and history of kidney and liver diseases (Wilson’s disease, autoimmune liver disease, virus infection, hemochromatosis, alcoholic fatty liver (, cardiovascular disease (CVD), diabetes, malignancy, thyroid disorder, and autoimmune. Also, the study did not include individuals who used potentially hepatotoxic or steatogenic drugs. Subjects with under-reported or over-reported dietary intake (≤ 800 or ≥ 4500 kcal/d) and those who completed less than 35 items of the food frequency questionnaire were excluded (8 participants) and were replaced. Written informed consent was obtained from all participants before the study enrollment.

### Dietary assessment

A valid and reliable 168-item semiquantitative food frequency questionnaire (FFQ) was used to collect participants’ dietary intakes [18]. FFQ has listed a collection of typical Iranian food with standard serving sizes [19]. We asked participants to explain their mean dietary intake over the past year by selecting one of the following categories: never or less than once a month, 3–4 times per month, once a week, 2–4 times per week, 5–6 times per week, once daily, 2–3 times per day, 4–5 times per day, and 6 or more times a day.

Portion sizes of each food item were transformed into grams using standard Iranian household measures. The daily intake of energy and nutrient for each person was calculated using the United States Department of Agriculture’s (USDA) Food Composition Table (FCT) [20]. We used Iranian FCT for some traditional food that did not exist in USDA FCT [21]. Then we converted consumed food frequency into a daily intake scale.

### AAs intake. AAs groups

Amino acids intake was calculated using USDA (USDA National Nutrient Database for Standard Reference, Release 28) FCT of 2015 (http://www.ars.usda.gov/ba/bhnrc/ndl), which is based on the chemical analyses of amino acids composition of over 5000 food items from all food groups. In the present study, we used three groups of amino acids, including BCAAs (leucine, isoleucine, and valine), AAAs (phenylalanine, tyrosine, and tryptophan), and SAAs (methionine and cysteine).

### Assessment of other variables

A nutritionist measured anthropometric indicators, including weight, height, and body mass index (BMI). A standard demographic questionnaire was used to assess age, sex, marital status, socioeconomic status (SES), and smoking status. We computed SES score according to three variables, including family size (≤ 4, > 4 people), education level (academic and non-academic), and acquisition (house ownership or not). For each variable, individuals were given a score of 1 (if their family had ≤ 4 members, had academic education or owned a house) or 0 (if their family had > 4 member, or had not academic education, or leasehold property). The total SES score was calculated through summing the assigned scores (minimum of 0 to maximum of 3). Participants with the score of 3, 2, and sum of subjects with 1 and 0 were classified as high, moderate, and low SES, respectively [22].

The short version of the International Physical Activity Questionnaire (IPAQ) measured physical activity through face-to-face interviews. All results of the IPAQ were reported as Metabolic Equivalents per week (METs/week). The details of all assessments were expressed in our previous study [22].

### Statistical analysis

Data analyses were conducted using Statistical Package for Social Sciences (version 20.0; SPSS Inc, Chicago, IL). We used Kolmogorov-Smirnov analysis and histogram charts to assess the normality of variables. Participants’ characteristics were expressed as mean ± SD or median (25–75 interquartile range) for continuous variables and percentages for categorical variables across tertiles of total dietary protein.

Chi-square and linear regression were used to test the trend of qualitative and quantitative variables across tertiles of dietary protein intake (as a median value in each tertile), respectively. Amino acid group intakes were computed as the percentage of total protein intake and categorized into tertiles based on their cut points among controls. Multivariable logistic regression models were used to assess the association between the tertiles of BCAAs, AAAs, and SAAs intake with the odds of NAFLD among the study participants. The odds ratio (OR) and 95% confidence interval (CI) were reported. Logistic regression models were adjusted for age, sex, BMI, physical activity, smoking, SES, dietary intake of energy, dietary fat, and fiber. P-values < 0.05 were considered statistically significant.

## Results

The mean ± standard deviation of age and BMI of participants (53% male) were 38.1 ± 8.8 years and 26.8 ± 4.3 kg/m2, respectively.

Participants’ demographic characteristics and dietary intake according to tertiles of dietary protein intake are shown in Table 1. Dietary intakes of energy, carbohydrate, fat, polyunsaturated fatty acids (PUFA), and fiber were decreased across tertiles of dietary protein intakes. However, BCAAs, AAAs, and SAAs were increased among tertiles of dietary protein intakes. There were no significant differences across tertiles of dietary protein intakes for other variables.

**Table 1:** Characteristics and dietary intakes across tertiles of dietary protein among the study participants

	**Tertiles of dietary protein (% of energy)**			***P*** **-trend**
	**T1 (** ***n*** ** = 232)**	**T2 (** ***n*** ** = 225)**	**T3 (** ***n*** ** = 218)**	
**Demographic variables**				
Age(year)	37.6 ± 8.5	38.4 ± 8.7	38.5 ± 9.4	0.258
Male, n (%)	120 (51.7)	131 (58.2)	107 (49.1)	0.137
BMI(Kg/m^2^)	26.9 ± 4.5	26.5 ± 4.0	27.2 ± 4.5	0.444
Smoking, n (%)	7 (3)	11 (4.9)	10 (4.6)	0.544
Physical activity (MET/min/week)	1424 ± 916	1496 ± 909	1377 ± 809	0.553
SES, n (%)				0.530
Low	80 (34.5)	64 (28.4)	79 (36.2)	
Middle	103 (44.4)	108 (48)	99 (45.4)	
High	49 (21.1)	53 (23.6)	40 (18.3)	
**Nutrient intake**				
Energy intake (Kcal/d)	2333 ± 655	2281 ± 642	2206 ± 619	0.035
Carbohydrate (% of energy)	57.8 ± 8.4	58.4 ± 6.4	55.8 ± 6.3	0.003
fat (% of energy)	33.3 ± 8.3	30.6 ± 6.3	30.8 ± 5.6	< 0.001
Saturated fats(% of energy)	10.1 ± 3.3	10.4 ± 2.8	10.6 ± 2.8	0.068
Polyunsaturated fatty acids(% of energy)	7.6 ± 2.8	6.0 ± 1.7	5.7 ± 1.6	< 0.001
Fibre (g/1000 kcal)	16.4 ± 8.5	16.8 ± 7.3	15.3 ± 5.2	0.014
BCAAs (% of energy)	1.99 ± 0.31	2.41 ± 0.32	2.83 ± 0.47	< 0.001
AAAs (% of energy)	1.03 ± 0.15	1.24 ± 0.15	1.45 ± 0.23	< 0.001
SAAs (% of energy)	0.41 ± 0.06	0.48 ± 0.06	0.57 ± 0.10	< 0.001

Linear regression analysis was used for obtaining *P* for trends

*SES* Socioeconomic status, *AAAs* Aromatic amino acids, *SAAs* Sulfuric amino acids, *BCAAs* Branched-chain amino acids

Table 2 demonstrates participants’ baseline characteristics and dietary intake in the case and control groups. Individuals in the NAFLD group had significantly higher BMI (< 0.001) and were more likely to be smokers (< 0.05). Also, the intake of energy (< 0.05), BCAAs, AAAs, and SAAs (< 0.001) were higher in cases than in controls. In contrast, people in the control group have higher physical activity compared with cases (< 0.001). Other characteristics did not significantly differ between the case and control groups.

**Table 2:** Characteristics and dietary intakes among cases and controls

	**Cases (** ***n*** ** = 225)**	**Controls (** ***n*** ** = 450)**	***P*** **-value**
**Demographic variables**			
Age(year)	38.63 ± 8.71	37.88 ± 8.92	0.293
Male, n (%)	125 (55.6)	233 (51.8)	0.354
BMI(Kg/m^2^)	30.56 ± 4.02	24.99 ± 3.10	< 0.001
Smoking, n (%)	16 (7.1)	12 (2.7)	0.008
Physical activity (MET/min/week)	1119 ± 616	1590 ± 949	< 0.001
SES, n (%)			0.127
Low	65 (28.9)	158 (35.1)	
Middle	104 (46.2)	206 (45.8)	
High	56 (24.9)	86 (19.1)	
**Nutrient intake**			
Energy intake (Kcal/d)	2369 ± 621	2227 ± 645	0.006
Carbohydrate (% of energy)	57.27 ± 7.69	57.37 ± 6.93	0.874
Protein (% of energy)	13.55 ± 2.55	13.60 ± 2.28	0.792
fat (% of energy)	31.49 ± 7.54	31.62 ± 6.64	0.823
Saturated fats(% of energy)	10.22 ± 3.07	10.49 ± 2.91	0.269
Polyunsaturated fatty acids(% of energy)	6.60 ± 2.47	6.35 ± 2.20	0.211
Fibre (g/1000 kcal)	16.77 ± 8.34	15.86 ± 6.47	0.013
BCAAs (% of energy)	2.51 ± 0.52	2.35 ± 0.49	< 0.001
AAAs (% of energy)	1.29 ± 0.25	1.21 ± 0.24	< 0.001
SAAs (% of energy)	0.51 ± 0.10	0.47 ± 0.09	< 0.001

*P*-values was calculated using independent sample t-test analysis for quantitative variables and chi-square test for qualitative variables

*SES* Socioeconomic status, *AAAs* Aromatic amino acids, *SAAs* Sulfuric amino acids, *BCAAs* Branched-chain amino acids

The percent of amino acid groups in main dietary sources in case and control groups are presented in Table 3. Participants in the control group had a significantly higher percentage of BCAAs, AAAs, and SAAs intake from vegetables (< 0.001) than the case group.

**Table 3:** The percent of amino acid groups in main dietary sources

Protein sources	Cases	Controls	*P*-Value
**BCAAs**			
Dairy	36.54 ± 12.46	36.86 ± 13.41	0.751
Cereals	26.71 ± 12.28	26.09 ± 11.52	0.531
Vegetables	2.77 ± 1.42	3.57 ± 1.84	< 0.001
White meat	12.58 ± 8.66	13.45 ± 8.63	0.220
Nuts	1.58 ± 2.21	1.69 ± 2.32	0.523
Red and process meat	8.11 ± 5.61	7.96 ± 6.22	0.762
Legume	2.13 ± 3.30	2.38 ± 3.32	0.354
**AAAs**			
Dairy	34.43 ± 12.20	34.77 ± 13.06	0.743
Cereals	29.24 ± 12.94	28.66 ± 12.13	0.577
Vegetables	2.98 ± 1.54	3.88 ± 1.99	< 0.001
White meat	11.74 ± 8.29	12.53 ± 8.22	0.241
Nuts	1.61 ± 2.20	1.74 ± 2.33	0.500
Red and process meat	7.57 ± 5.33	7.43 ± 5.85	0.757
Legume	2.26 ± 3.49	2.54 ± 3.59	0.322
**SAAs**			
Dairy	29.02 ± 11.25	29.28 ± 12.05	0.799
Cereals	31.48 ± 13.37	30.95 ± 12.48	0.623
Vegetables	2.89 ± 1.58	3.82 ± 2.10	< 0.001
White meat	14.16 ± 9.48	15.10 ± 9.38	0.222
Nuts	1.29 ± 1.78	1.39 ± 1.92	0.474
Red and process meat	8.32 ± 5.82	8.16 ± 6.30	0.748
Legume	1.62 ± 2.61	1.83 ± 2.72	0.342

*P*-values was calculated using independent sample t-test analysis

^*^ Data presented as mean ± SD

Table 4 indicates the ORs and 95% CI of NAFLD across tertiles of AAs. In the crude model, participants who were in the highest tertiles of BCAAs (OR = 2.58; 95% CI: 1.67–3.99, *P* for trend = < 0.001)), AAAs (OR = 2.58; %95CI: 1.67–3.99, *P* for trend = < 0.001)) and SAAs (OR = 2.77; 95% CI: 1.78–4.31, P for trend = < 0.001)) intakes had a higher odd of NAFLD compared to those in the lowest tertile.

**Table 4:** Odds ratios (ORs) and 95% confidence intervals (CIs) for NAFLD based on tertiles amino acids

	**Tertiles of dietary intake**			***P*** **-trend**
**Amino acid groups**	**T1**	**T2**	**T3**	
**BCAAs**				
Median score				
NAFLD /control	38 / 149	85 / 146	102 / 155	
Crude model	1.00 (Ref)	2.28 (1.46—3.56)	2.58 (1.67—3.99)	< 0.001
Model 1^a^	1.00 (Ref)	2.28 (1.46—3.56)	2.55 (1.65—3.95)	< 0.001
Model 2 ^b^	1.00 (Ref)	2.67 (1.42—5.01)	2.58 (1.40—4.76)	0.009
Model 3^c^	1.00 (Ref)	2.83 (1.49—5.38)	2.82 (1.50—5.30)	0.005
**AAAs**				
Median score				
NAFLD /control	38 / 149	85 / 146	102 / 155	
Crude model	1.00 (Ref)	2.28 (1.46—3.56)	2.58 (1.67—3.99)	< 0.001
Model 1^a^	1.00 (Ref)	2.28 (1.46—3.56)	2.55 (1.65—3.95)	< 0.001
Model 2 ^b^	1.00 (Ref)	2.67 (1.42—5.01)	2.58 (1.40—4.76)	0.008
Model 3^c^	1.00 (Ref)	2.83 (1.49—5.38)	2.82 (1.50—5.30)	0.005
**SAAs**				
NAFLD /control	36 / 149	88 / 150	101 / 151	
Crude model	1.00 (Ref)	2.43 (1.55—3.81)	2.77 (1.78—4.31)	< 0.001
Model 1^a^	1.00 (Ref)	2.39 (1.52—3.76)	2.72 (1.74—4.25)	< 0.001
Model 2 ^b^	1.00 (Ref)	3.02 (1.59—5.77)	2.59 (1.38—4.85)	0.017
Model 3^c^	1.00 (Ref)	3.19 (1.66—6.15)	2.86 (1.49—5.48)	0.010

*P* for trends was calculated using logistic regression analysis

*AAAs* Aromatic amino acids, *SAAs* Sulfuric amino acids, *BCAAs* Branched-chain amino acids

^a^ Adjusted for age and sex

^b^ Adjusted for model 1 and BMI, physical activity, smoking, SES, dietary intake of energy

^c^ Adjusted for model 2 and dietary fat and fiber

The association between AAAs, BCAAs, and SAAs with odds of NAFLD remained significant in all adjusted models. In the final model, adjusted for age, sex, BMI, PA, smoking status, SES, dietary intake of energy, dietary fat, and fiber, the OR (95% CI) of NAFLD were (OR = 2.82; 95% CI: 1.50–5.30, *P* for trend = 0.005), (OR = 2.82; 95% CI: 1.50–5.30, P for trend = 0.005), (OR = 2.86; 95% CI: 1.49–5.48, *P* for trend = 0.010) for the highest vs. lowest tertiles of BCAAs, AAAs, and SAAs, respectively.

## Discussion

The present case-control study among Iranian adults indicated that higher intakes of BCAAs, AAAs, and SAAs are related to increased odds of NAFLD.

In line with our results, a previous study by Galarregui et al. indicated that higher dietary intakes of BCAAs, AAAs, and SAAs, were significantly associated with higher liver fat mass among adults adjusted for potential confounders [17].

Based on our knowledge, studies about dietary AAAs and SAAs relationship with chronic disease are scarce. However, several studies investigated the BCAAs with different chronic diseases [11]. Despite some inconsistent results, more epidemiological studies suggest that a high dietary intake of BCAAs is associated with a higher risk of type 2 diabetes (T2D) [23]. Also, recent observational studies reported that a higher intake of BCAAs may elicit insulin resistance and glucose intolerance [10, 24, 25]. The results of BCAAs’ relation to obesity are inconsistent. Some studies showed a positive association between BCAAs and obesity [26, 27], and some showed a negative association [24, 25]. controversial results may be related to differences in study characteristics, including design, sample size, population groups, race, genetic factors, dietary habits, nutritional culture, and various food sources. In a study by Zheng et al., dietary intake of BCAA increased the risk of diabetes by %13 in US adults [28]. However, Nagata et al. showed a beneficial effect of dietary BCAA in preventing diabetes among Japanese adults [29]. One main justification for these inconsistent results attributed to the food sources of BCAAs among the US and Japanese population, which was cereals, potatoes, starches, fish, and meats among Japanese, whereas, in the US diet, BCAAs is more supplied by meats, fish, and milk, respectively. In our study, the main source of BCAAs, AAAs, and other AAs, respectively, was dairy products, cereal, and meats, closer to the US diet.

Similar to our results, one previous study showed a direct relationship between dietary SAAs and cysteine with a higher prevalence of Overweight/Obesity, BMI and waist circumference, unfavorable lipid profiles, and IR in Northern Chinese adults [15]. Another study reported that a high-SAA diet is associated with an increased risk for diabetes mortality [30]. Also, Dong et al. indicate that higher intake of SAA, Methionine, and Cysteine, independent of protein intake, was associated with significant increases in cardiometabolic disease risk factors, including serum cholesterol, glucose, uric acid, BUN, insulin, and glycated hemoglobin [31]. In another study, SAAs showed a positive correlation with total cholesterol(TC) and triglycerides(TGs) and a negative correlation with high-density lipoprotein cholesterol (HDL-c) [32].

To our knowledge, except for a study by Galarregui et al., AAAs were not previously investigated with fatty liver in epidemiologic studies. However, a previous study among Iranian adults showed a significant positive association between AAAs and hypertension [33]. AAAs components, including phenylalanine, tyrosine, and tryptophan, were investigated with cardiometabolic factors in previous studies [32, 34]. In a study by Lieberman et al., tryptophan intake, as a component of AAAs, was not related to most markers of liver function among US adults [34]. In another study, dietary tryptophan was negatively related to TC and TGs. However, tyrosine showed a positive association with TGs [32].

Studies have shown that the dietary intake of AAs affected their serum level. Two studies reported that %80 of serum BCAA concentration is determined by dietary intake of BCAA and the remaining 20% consists of the catabolism of BCAA metabolites [35, 36]. Most studies on serum BCAAs, demonstrate a negative relation between circulating BCAAs levels and chronic diseases such as hyperlipidemia, obesity, IR, T2D, and cardiovascular diseases [23, 37–40]. Also, studies indicated that an alteration in BCAA metabolism might be a pathological condition of NAFLD, and higher levels of BCAAs are associated with the heterogeneity of hepatic steatosis in patients with NAFLD [41, 42].

Although mechanisms underlying the role of BCAAs, AAAs, and SAAs on the risk of NAFLD are unclear, some potential pathways have been proposed to explain how dietary BCAAs lead to IR, obesity, and T2D. It suggested that a high and chronic intake of BCAAs activates the mammalian target of rapamycin complex 1 (mTORC1), a critical cellular pathway involved in cell growth, differentiation, cell survival, and metabolism, leading to blocking insulin intracellular signaling and increasing IR. Also, it proposed that in individuals with impaired BCAAs metabolism, increases the level of toxic metabolites of BCAAs caused mitochondrial dysfunction of pancreatic $\beta$ cells and resulted in IR, obesity, and other related complications [10, 43, 44].

Another mechanism that may justify the NAFLD caused by the high intake of BCAAs, AAAs, and SAAs relates to those dietary sources of protein intake, mostly consumed from animal sources [31, 33, 45]. In our study, the main source of AAs groups was animal protein, including meats and high-fat dairy, which previously showed an adverse association with NAFLD [46, 47].

Our study has some advantages; contrary to some studies that used self-report questionnaires, in the present study, dietary data were filled out by expert interviewers in a face-to-face interview, using a validated and repeatable 168-item food frequency questionnaire (FFQ) [48], which lessens measurement bias. We do not rule out the possibility of other unknown confounders, so their effects may have occurred. Our study also has some limitations. Firstly, the present study cannot discover the causal relationship due to the case-control design. Secondly, the inevitable measurement error of FFQ may also affect our results. Thirdly, we did not have data on pubertal status, the number of pregnancies, hormonal conditions of participants, genetic data, and specialty circulating BCAAs, AAAs, and SAAs concentration for further investigations.

## Conclusion

Our study indicated a direct association between the intake of AAs groups, including BCAAs, AAAs, SAAs, and the odds of NAFLD. We suggest that other researchers examine the association between AAs groups and NAFLD in large cohort studies.

## Data Availability

The data analyzed in the present study are available by the corresponding author at a reasonable request.

## Abbreviations

NAFLD: Nonalcoholic fatty liver disease
AAs: amino acids
BCAAs: branched-chain amino acids
AAAs: aromatic amino acids
SAAs: sulfuric amino acids
IR: Insulin resistance
FFQ: Food Frequency Questionnaire
SES: Socioeconomic status
IPAQ: International Physical Activity Questionnaire
USDA: United States department of agriculture
FCT: Food composition table
BMI: Body mass index
TCA: tricarboxylic acid
NASH: nonalcoholic steatohepatitis
TC: total cholesterol
HDL-C: high-density lipoprotein cholesterol
TG: triglyceride
CVD: cardiovascular disease
T2D: type 2 diabetes
METs: Metabolic Equivalents
OR: odds ratio
CI: confidence interval
PUFA: polyunsaturated fatty acids
